# The Geriatric Nutrition Risk Index Is Not a Prognostic Predictor for Postoperative Morbidity in Extremely Elderly Patients Undergoing Surgery for Proximal Femur Fractures

**DOI:** 10.3390/jcm13216333

**Published:** 2024-10-23

**Authors:** Jung Ju Choi, Chun Gon Park, Ji Woong Kim, Youn Yi Jo

**Affiliations:** Department of Anesthesiology and Pain Medicine, Gachon University College of Medicine, Gil Hospital, Incheon 21565, Republic of Korea; jjchoi2@gilhospital.com (J.J.C.); chungony@gilhospital.com (C.G.P.); 19709@gilhospital.com (J.W.K.)

**Keywords:** geriatric nutrition risk index, extremely elderly, proximal femur fracture, morbidity

## Abstract

**Background/Objectives:** The geriatric nutrition risk index (GnRI) has been regarded as a useful predictor of morbidity and mortality in elderly patients. This study aimed to determine the use of the GnRI as a prognostic predictor in extremely elderly patients undergoing proximal femur fracture surgery and the usefulness of evaluation tools related to a patient’s underlying disease and functional capacity in predicting the prognosis of extremely elderly patients. **Methods**: We analyzed 548 patients who had undergone surgery for proximal femur fracture caused by trauma, with an age of ≥80 years, without other accompanying trauma. **Results**: Body mass index (BMI) (OR, 1.077; 95% CI, 1.010–1.149; *p* = 0.023), serum albumin levels (0.389; 0.223–0.678; *p* = 0.001), and Charlson comorbidity index (CCI) (1.170; 1.014–1.349; *p* = 0.031) were determined to be predictors of morbidity in a multivariable regression analysis. The area under the curve (AUC) in the receiver operating characteristic curve of BMI was 0.565 (95% CI, 0.493–0.637; *p* = 0.065), and the optimal cut-off value could not be determined. The AUC of serum albumin was 0.647 (0.576–0.717; *p* < 0.001), and the optimal cut-off value was 3.65 g/dL (sensitivity, 72.2%; specificity, 52.7%). The AUC of the CCI was 0.648 (0.580–0.715; *p* < 0.001), and the optimal cut-off value was 6.5 (sensitivity, 63.3%; specificity, 61.4%). **Conclusions**: The GnRI was not a predictive factor for patient prognosis after proximal femur fractures in extreme elderly patients. Rather, serum albumin level and CCI, which reflect the patient’s underlying comorbid conditions, were more useful in predicting in-hospital morbidity after proximal femur surgery in extremely elderly patients.

## 1. Introduction

Femur fractures are a major cause of hospital admissions in the elderly population and are associated with mortality, morbidity, and functional impairment [1]. In particular, the 1-year mortality rate for proximal femur fractures is approximately 30% [2]. Femur fractures are closely associated with frailty in the elderly. Osteoporosis, which is frequent in elderly people, and underlying comorbidities that contribute to frailty may lead to proximal femur fractures, even in cases of minor trauma [3]. Frailty is characterized by weakness, weight loss, decreased grip strength, and slow gait owing to increased vulnerability resulting from a decline in physical ability with aging [4]. In a previous review of 502 cases of proximal femur fractures, the mortality rate in the group with a high clinical frailty score was >10 times higher than that in the group with a low clinical frailty score (3.8% vs. 41.7%) [3].

The geriatric nutrition risk index (GnRI) is calculated from serum albumin levels and body weight and reflects frailty and loss of muscle mass [5]. In a previous analysis of 82,725 patients (aged > 65 years) who underwent emergency surgery, malnutrition with a low GnRI worsened the risk of mortality, morbidity, and prolonged hospital stay [6]. In other studies analyzing elderly trauma patients, the GnRI was an independent variable predicting mortality and length of hospital stay [7,8]. In a study by Huang et al. (2021), the patient group with a lower GnRI had 5.7-fold higher mortality (*p* = 0.003) and a longer hospital stay (*p* = 0.016) than the normal GnRI group in elderly patients with fall injuries.

Although recent studies define the oldest old age as over 85 or 90 years, the American Geriatric Society and the World Health Organization define the oldest old age as over 80 years, so this study was conducted on subjects aged over 80 years [9,10].

Although research results on the GnRI and prognosis of trauma patients have been recently published, there is no research on the GnRI and prognosis of extremely elderly patients who have undergone surgery for proximal femur fractures. We expected that extremely elderly patients with a low GnRI would have a poor prognosis after proximal femur fracture surgery. This study aimed to determine the relationship between the GnRI and prognosis in elderly patients undergoing proximal femur fracture surgery. Additionally, we aimed to determine the usefulness of evaluation tools related to a patient’s underlying disease and functional capacity in predicting the prognosis of extremely elderly patients.

## 2. Materials and Methods

### 2.1. Ethical Considerations and Participants

This retrospective study was approved by the Institutional Review Board of the Gachon University Gil Hospital. Of the 605 patients who had undergone surgery for proximal femur fracture caused by trauma, such as slipping, falling, and hitting, with an age of ≥80 years, from January 2020 to April 2024, we excluded 57 cases with pathologic fractures caused due to metastasis of malignancy, avascular necrosis, and severe trauma with other organ damage. Accordingly, the medical records of 548 patients who had undergone proximal femoral surgery were analyzed.

### 2.2. Analyzing Variables

The preoperative data included age, sex, weight, height, American Society of Anesthesiologists physical status (ASA PS), Charlson comorbidity index (CCI), clinical frailty scale (CFS), Koval index, and preoperative serum albumin levels. The ASA PS is an assessment tool that classifies the severity of a patient’s condition based on the patient’s medical condition before anesthesia. The CCI was calculated using the following factors: age, history of myocardial infarction, congestive heart failure, peripheral vascular disease, history of cerebrovascular accident with or without sequelae, dementia, chronic pulmonary disease, connective tissue disease, peptic ulcer disease, severity of liver disease, diabetes with or without complications, hemiplegia, severity of chronic kidney disease, solid tumors with or without metastasis, hematologic malignancy, and acquired immunodeficiency syndrome; these were weighted according to their influence on mortality. The CFS is a tool for assessing frailty in the elderly over 65 years of age (score from 1 to 9) and includes mobility and balance with or without walking aids and the ability to perform activities of daily living. The Koval score is a tool for assessing mobility (scores from 0: non-ambulators to 5: independent community ambulators) [11]. The GnRI was calculated using the following equation: GnRI = (1.489 × serum albumin on admission [g/L]) + (41.7 × [weight/ideal body weight]); ideal body weight was calculated as follows: ideal body weight = (height − 100 − [height − 150]/[4 in males and 2 in females]).

Morbidity was defined as a disease acquired after femur surgery that seriously affected the patient’s vital signs, including death, pneumonia, severe pulmonary edema, massive pulmonary thromboembolism, newly acquired arrhythmia, aggravation of heart failure, acute coronary syndrome, acute kidney injury with a 2-fold increase in serum creatinine levels from baseline, gastrointestinal bleeding, and cerebrovascular attack. Based on the definition of morbidity above, we performed intergroup analysis of patient characteristics and preoperative data between the non-morbidity group and the morbidity group.

The primary endpoint of this study was to determine the relationship between the GnRI and postoperative morbidity in extremely elderly patients after proximal femur surgery, and the secondary endpoint was to determine the relationship between health-related indicators and morbidity. Based on previous research in elderly trauma patients where the overall mean GnRI was 93.4, and the GnRI in the deceased group was 89.8 ± 12.9, the sample size was calculated to be 548 with a power of 90% and an error of 0.05 [12].

### 2.3. Statistical Analysis

SPSS ver. 22 (SPSS Inc., Chicago, IL, USA) was used for data analysis. To assess the normality of data, a Kolmogorov–Smirnov test was used. For comparisons of the differences between the non-morbidity and morbidity groups, we used an independent *t*-test or Mann–Whitney U test for continuous data, and a Chi-square test for categorical data. The results are presented as mean ± standard deviation, median (interquartile range), or number of patients. To confirm the independent risk factors of morbidity endpoints, binary logistic regression analysis was performed. Factors with *p* < 0.05 in the univariable regression analysis were analyzed using multivariable regression analysis. Receiver operating characteristic (ROC) curves were used to determine the ability of the variables to predict major morbidities. The point on the ROC curve where specificity and sensitivity were maximized was set as the cut-off value. Statistical significance was set at *p* < 0.05 significant.

## 3. Results

The patient characteristics and preoperative data are presented in Table 1. The overall in-hospital morbidity rate was 14.4% (79 of 548 patients) and the overall in-hospital mortality rate was 4.0% (22 of 548 patients). The mean age was 86 ± 4. There were more patients with ASA PS ≥ 3 in the morbidity group than in the non-morbidity group (75.9% vs. 58.2%, *p* = 0.001). There were statistically significant differences in body mass index (BMI) and preoperative serum albumin levels (*p* = 0.032 and <0.001, respectively). However, the GnRI was similar between the groups (*p* = 0.226). The CCI was significantly higher in the morbidity group than in the non-morbidity group (*p* < 0.001). Postoperative hospital stay was 10.4 ± 5.2 days in the non-morbidity group and 19.5 ± 13.2 days in the morbidity group (*p* < 0.001).

The postoperative morbidity data are shown in Table 2 (in some cases, one patient may have multiple morbidities). The morbidity incidence was 18.3% (21/115) in men and 13.4% (58/433) in women (*p* = 0.183). Of the 79 morbidity patients, 22 died. The most common causes of death were pneumonia in 11 cases (acute respiratory distress syndrome or septic shock due to pneumonia), cardiogenic shock in 5 cases, gastrointestinal origin in 4 cases (2 biliary sepsis, 1 hepatic encephalopathy, and 1 panperitonitis), and acute kidney injury in 1 case. The mean GnRI values of each major morbidity (death, respiratory, cardiac, renal, gastrointestinal, cerebral) were 94 ± 12, 93 ± 10, 95 ± 11, 99 ± 13, 94 ± 13, and 97 ± 10, respectively.

The results of the logistic regression analysis to identify the factors associated with major morbidities are shown in Table 3. There were no missing data for any of the variables. All the variables listed in Table 1 were included in the logistic regression analysis. Of the variables, BMI, ASA PS, serum albumin levels, and CCI had *p* values < 0.05 in the univariable regression analysis, and in the multivariable regression analysis, BMI (OR, 1.077; 95% CI, 1.010–1.149; *p* = 0.023), serum albumin levels (0.389; 0.223–0.678; *p* = 0.001), and CCI (1.170; 1.014–1.349; *p* = 0.031) were determined to be predictors of morbidity.

When the GnRI values were divided into <82, 82–91, 92–98, and 98< and the association with each frequency and morbidity was further analyzed, it was 40.1% (188/469), 22.0% (103/469), 30.5% (143/469), and 7.5% (35/469) in the non-morbidity group and it was 34.2% (27/79), 16.5% (13/790, 38.0% (30/79), and 11.4% (9/79) in the morbidity group (*p* = 0.254), and in the univariable regression analysis, the OR was 1.228 (95% CI 0.972–1.550; *p* = 0.085).

In a subgroup analysis of the variables in Table 1 by sex (Table 4), only CCI (1.294; 1.022–1.639; *p* = 0.032) in males and BMI, ASA, serum albumin levels, and CCI in females had *p* < 0.05, as determined by the univariable regression analysis. In the multivariable regression analysis, BMI (1.113; 1.031–1.202; *p* = 0.006) and serum albumin levels (0.294; 0.154–0.564; *p* < 0.001) were statistically significant predictors of morbidity in females. However, the GnRI calculated based on BMI and serum albumin levels showed no significant findings in either males or females.

**Figure 1:**
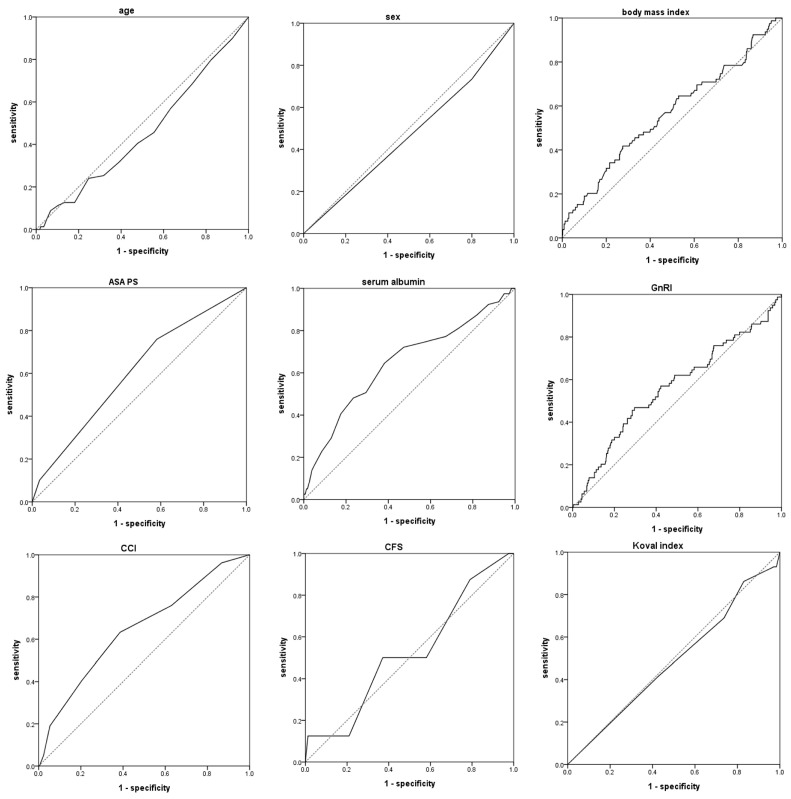
Receiver operating characteristic curves of age, sex, body mass index, ASA PS, serum albumin, GnRI, CCI, CFS, and Koval index. ASA PS, American Society of Anesthesiologists physical status; GnRI, geriatric nutrition risk index; CCI, Charlson comorbidity index; CFS, clinical frailty scale.

We analyzed all variables applied to the regression analysis using the ROC curve (Figure 1). The area under the curve (AUC) of age, sex, BMI, ASA PS, serum albumin, the GnRI, CCI, CFS, and the Koval index were 0.457 (95% CI 0.388–0.526; *p* = 0.223), 0.467 (95% CI 0.397–0.538; *p* = 0.352), 0.565 (95% CI 0.493–0.637; *p* = 0.065), 0.605 (95% CI 0.539–0.672; *p* = 0.003), 0.647 (95% CI 0.576–0.717; *p* < 0.001), 0.561 (95% CI 0.488–0.634; *p* = 0.083), 0.648 (95% CI 0.580–0.715; *p* < 0.001), 0.523 (95% CI 0.321–0.725; *p* = 0.828), and 0.486 (95% CI 0.373–0.599; *p* = 0.808), respectively. The optimal cut-off values of significant variables (ASA PS, serum albumin, and CCI) were 3 (sensitivity, 75.9%; specificity, 51.8%), 3.65 g/dL (sensitivity, 72.2%; specificity, 52.7%), and 6.5 (sensitivity, 63.3%; specificity, 61.4%), respectively. The optimal cut off value for the GnRI was not derived.

**Figure 2:**
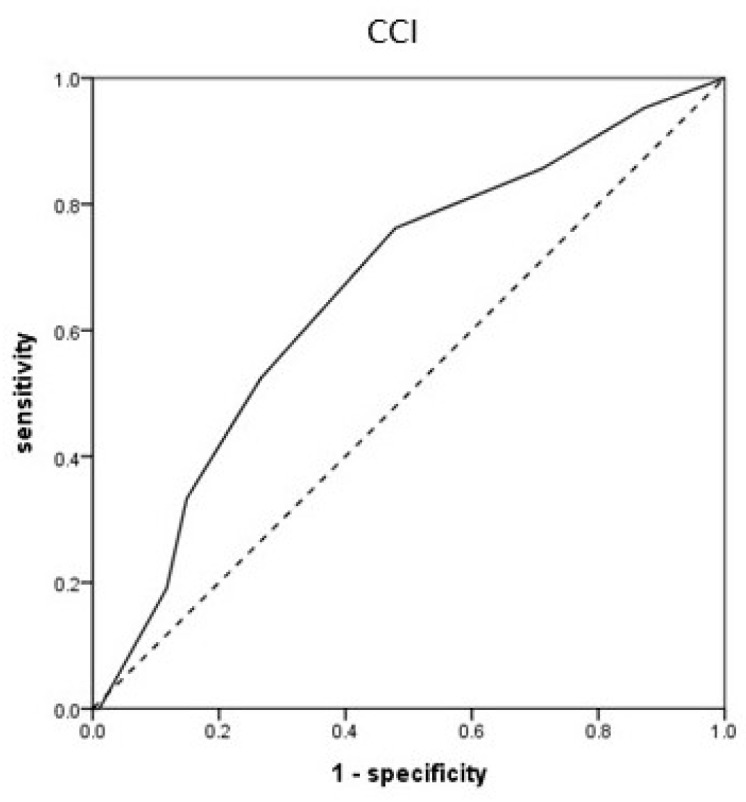
Receiver operating characteristic curves of Charlson comorbidity index (CCI) in males.

**Figure 3:**
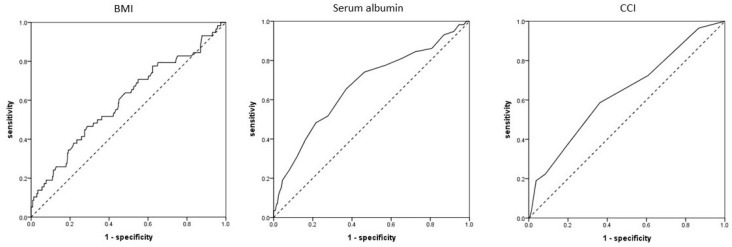
Receiver operating characteristic curves of body mass index (BMI), serum albumin levels, and Charlson comorbidity index (CCI) in females.

In males, the AUC of the CCI was 0.664 (0.537–0.790; *p* = 0.019), and the optimal cut-off value was 6.5 (sensitivity, 76.2%; specificity, 52.1%) (Figure 2). In females, the AUC of BMI was 0.598 (95% CI 0.515–0.681; *p* = 0.016), and the optimal cut-off value was 22.3 kg/m^2^ (sensitivity, 58.6%; specificity, 55.2%). The AUC of serum albumin levels was 0.670 (0.590–0.749, *p* < 0.001), and the optimal cut-off value was 3.55 g/dL (sensitivity, 65.5%; specificity, 62.9%). The AUC of the CCI was 0.636 (0.557–0.715; *p* = 0.001), and the optimal cut-off value was 6.5 (sensitivity, 58.6%; specificity, 63.7%) (Figure 3).

## 4. Discussion

In extremely elderly patients who had undergone surgery for proximal femur fractures, the GnRI was not found to be a predictive factor for in-hospital morbidity after surgery. Serum albumin levels and body weight were positively correlated in the GnRI calculation; however, in this study, low albumin levels and high BMI were associated with morbidity. The CCI, which is related to a patient’s underlying comorbid conditions, was found to be a more useful factor in predicting in-hospital morbidity after proximal femur surgery in extremely elderly patients.

A previous retrospective analysis of 2430 patients reported that in elderly patients (aged > 65 years), the frequency of femur fractures was 2.9 times higher in women than in men, and they concluded that the decrease in bone mass in women may have influenced these results [13]. Another study found that the prevalence of osteoporosis in people over 80 years of age was 26.7% in men and 85.8% in women [14]. It is thought that the fact that there were more female patients than male patients in our study may be due to this influence.

The GnRI is closely related to frailty [5]. An analysis of 740 elderly patients (aged > 70 years) revealed that GnRI levels were independently associated with frailty (OR, 0.95; 95% CI 0.93–0.97; *p* < 0.001) [5]. Other studies have shown that CFS is a useful tool for predicting mortality after proximal femoral fracture surgery [3]. However, our study failed to demonstrate a correlation between each tool and the patient’s postoperative prognosis, not only in the frailty score itself, but also in the GnRI, which reflects frailty and nutritional status. However, because our study only targeted extremely elderly patients aged ≥ 80 years, our results may differ from those of previous studies. Also, in a previous study of other elderly trauma [8], the proportion of patients belonging to the no-risk group with a GnRI value of 98 or higher was 68.4%, but in our study, it was less than 10% (44/548). This is thought to be because we only targeted patients with proximal femur fractures among trauma patients. A recent study of 580 patients demonstrated that a low GnRI was closely related to fragility fractures [15]. The prevalence of frailty varies greatly depending on the country, from 20% to over 80%, and increases sharply as the elderly population reaches extreme old age [16]. According to a previous study analyzing 13,859 participants (mean age: 85.8 ± 11.1 years), non-frailty was 54.3% at 65–79 years, 26.9% at 80–89 years, 15% at 90–99 years, and 3.8% at ≥100 years of age; thus, even in the elderly, increasing age shows a close relationship with increasing frailty [17]. The GnRI is positively correlated with serum albumin levels and body weight. However, in a previous meta-analysis that included 7751 obese patients, obesity increased the risk of mortality, morbidity, and ICU stay, with a longer duration of mechanical ventilator application after trauma [18]. This is in conflict with our research results on the GnRI. In our study, BMI was significantly higher but serum albumin levels were significantly lower in the morbidity group than in the non-morbidity group. These results led to the finding that there was no statistically significant difference in the GnRI between the two groups.

In this study, the CCI was a predictor of prognosis after proximal femoral fracture surgery in extremely elderly patients. According to a previous study that analyzed the outcomes of trauma patients by adding the CCI to the Trauma and Injury Severity Score (TRISS) in 2819 major trauma patients, the CCI appeared to be useful in predicting death (*p* < 0.001), but adding the CCI to the TRISS had no additional benefit compared with the TRISS alone [19]. Even in the results of the analysis considering age, adding the CCI to the TRISS did not improve outcome prediction compared with the TRISS alone [20]. Because our study only targeted patients who did not have severe damage to other body parts, except for proximal femur fractures, it appears that the patient’s underlying disease was more closely related to prognosis than to the severity of trauma. In another study that analyzed only a part of spinal trauma, the injury severity score did not show a correlation with mortality, but the CCI showed a close relationship with mortality [19].

Conflicting results have been published on the relationship between BMI and patient prognosis [21,22]. A study analyzing 182 elderly patients reported that mortality increased when BMI was <18.5 kg/m^2^ and albumin was <2.8 g/dL [21]. In contrast, another meta-analysis showed that for BMI > 40 kg/m^2^, mortality after trauma increased, and the length of hospital and ICU stays also increased [22]. In our study, BMI was analyzed as one of the factors that increased morbidity; however, this does not mean that it falls under the definition of underweight or obesity as in previous studies. In addition, the average BMI in both the morbidity and non-morbidity groups was within the normal range, and the increases and decreases in BMI and serum albumin levels were opposite in the two groups; therefore, it is difficult to compare and reflect the results of previous studies with those of our study.

Serum albumin is a factor that reflects a patient’s nutritional status and affects postoperative prognosis [23], and low serum albumin levels are associated with poor outcomes in acute inflammation and disease [24], showing that the underlying disease, regardless of its correlation with albumin levels, is thought to be useful in predicting patient prognosis. Some researchers have focused on the role of albumin as part of a prognostic index, such as the CCI, rather than serum albumin alone [25]. We used the ROC curve to obtain the optimal cutoff value of serum albumin for predicting major morbidity, and the result was 3.55 g/dL. This result is consistent with previous studies showing that in-hospital mortality increases in cases of hypo-albuminemia with serum albumin levels below 3.5 g/dL [26]. Serum albumin level (<3.5 g/dL) on admission was reported to be a useful factor in predicting 1-year mortality in patients undergoing surgery for femur neck fracture (*p* = 0.0049) [27]. However, although we decided on the cut-off value as the point where sensitivity and specificity are maximized on the ROC curve, it is difficult to make a decision based on this threshold value because the AUC of each variable is less than 0.7 and we targeted short-term (in-hospital) major morbidity rather than mortality. Several studies show that changes in serum albumin during hospital stay are associated with poor outcomes in various diseases, such as cancer, end-stage renal disease, cardiologic disease, and traumatic brain injury [28,29,30,31]. Because we analyzed only the relationship between preoperative serum albumin and morbidity, not the change in albumin levels during the hospital stay, there are limitations in generalizing the relationship between serum albumin and prognosis in elderly trauma patients.

Sex differences can lead to differences in morbidity and mortality rates [32,33]. In a recent retrospective analysis of 4432 geriatric trauma patients (1635 males and 3859 females), male sex (OR, 1.94; 95% CI, 1.38–2.73; *p* < 0.001) was an independent mortality predictor of injury severity score, shock on admission in the emergency room, and underlying comorbidity in geriatric patients (mean age: 81 ± 8.5) [33]. We performed a subgroup analysis by sex to determine whether factors predicting patient prognosis differed depending on sex in patients with isolated femoral fractures without accompanying injuries, which are more frequently experienced by the elderly. Consequently, the CCI was identified as an independent mortality predictor in men, and serum albumin levels and BMI were identified as mortality predictors in in women. A previous study on patients with myocardial infarction reported that serum albumin levels were associated with increased mortality only in women [34]. Therefore, further research is required to determine the use of serum albumin levels and sex differences.

Our study had several limitations. First, because we only analyzed morbidity as an endpoint, there may be differences in patients’ quality of life or return to daily life after surgery. Second, the possibility that some factors suspected to have occurred after surgery in this study may have caused the patients’ trauma cannot be ruled out. For example, sudden motor weakness due to CVA or worsening of the general condition due to pneumonia may have caused slipping and led to a femur fracture. Third, because we targeted Asian patients, it is difficult to apply our results to all ethnicities. Lastly, selection bias could not be avoided, because cases in which morbidity occurred after transfer to another hospital or nursing home during treatment were omitted. In addition, cases that did not develop symptoms during the hospitalization period but were readmitted or treated at another hospital due to morbidity after discharge may have been omitted, which may have led to selection bias and confusion between the non-morbidity and morbidity groups, and thus, there is a possibility of misclassification bias.

## 5. Conclusions

In conclusion, the GnRI was not a predictive factor for patient prognosis after proximal femur fractures in extremely elderly patients. Rather, serum albumin levels and CCI, which reflect a patient’s underlying comorbid conditions, were more useful in predicting in-hospital morbidity after proximal femur surgery in extremely elderly patients.

## Figures and Tables

**Table 1:** Patient characteristics and preoperative data.

	Overall n = 548	Non-Morbidity n = 469	Morbidity n = 79	*p*-Value
Age, years	86 ± 4	86 ± 4	85 ± 4	0.238
Sex, M/F	115/433	94/375	21/58	0.183
BMI, kg/m^2^	22 ± 4	22 ± 4	23 ± 4	0.032
ASA PS, 2/3/4	215/309/24	196/257/16	19/52/8	0.001
Serum albumin (g/dL)	3.6 ± 0.5	3.7 ± 0.4	3.4 ± 0.5	<0.001
GnRI	96 ± 10	96 ± 10	94 ± 11	0.226
CCI	6 (5–7)	6 (5–7)	7 (6–9)	<0.001
CFS	4 (3–5)	4 (3–5)	4 (3–5)	0.714
Koval index	4 (3–5)	4 (3–5)	4 (3–5)	0.620
Postop hospital stay, days	11.7 ± 7.6	10.4 ± 5.2	19.5 ± 13.2	<0.001

Values are presented as mean ± SD, number of patients, or median (interquartile range). BMI, body mass index; ASA PS, American Society of Anesthesiologists physical status; GnRI, geriatric nutrition risk index; CCI, Charlson comorbidity index; CFS, clinical frailty scale.

**Table 2:** Postoperative morbidity data.

	Major Morbidity (n = 79)
Death	22 (27.8)
Respiratory	41 (51.9)
Cardiac	15 (19.0)
Renal	12 (15.2)
Gastrointestinal	13 (16.5)
Cerebral	6 (7.6)

Values are presented as number of patients (%).

**Table 3:** Logistic regression analysis to identify factors associated with major morbidities.

	Univariable			Multivariable		
	OR	95% CI	*p*-Value	OR	95% CI	*p*-Value
Age	0.966	0.910–1.024	0.247			
Sex	0.692	0.400–1.198	0.188			
BMI	1.082	1.017–1.150	0.012	1.077	1.010–1.149	0.023
ASA PS	2.198	1.418–3.408	<0.001	1.510	0.931–2.448	0.095
Serum albumin	0.318	0.189–0.535	<0.001	0.389	0.223–0.678	0.001
GnRI	0.986	0.963–1.009	0.226			
CCI	1.322	1.168–1.498	<0.001	1.170	1.014–1.349	0.031
CFS	1.083	0.709–1.652	0.713			
Koval index	0.926	0.685–1.252	0.619			

OR, odds ratio; 95% CI, 95% confidence interval; BMI, body mass index; ASA PS, American Society of Anesthesiologists physical status; GnRI, geriatric nutrition risk index; CCI, Charlson comorbidity index; CFS, clinical frailty scale.

**Table 4:** Risk factors associated with major morbidities according to sex.

	Univariable			Multivariable		
	OR	95% CI	*p*-Value	OR	95% CI	*p*-Value
**Male**						
GnRI	0.985	0.938–1.034	0.543			
CCI	1.294	1.022–1.639	0.032	1.294	1.022–1.639	0.032
**Female**						
BMI	1.115	1.040–1.196	0.002	1.113	1.031–1.202	0.006
ASA PS	2.421	1.463–4.008	0.001	1.675	0.950–2.951	0.074
Serum albumin	0.278	0.155–0.501	<0.001	0.294	0.154–0.564	<0.001
GnRI	0.988	0.962–1.015	0.395			
CCI	1.305	1.128–1.510	<0.001	1.104	0.927–1.315	0.268

OR, odds ratio; 95% CI, 95% confidence interval; BMI, body mass index; ASA PS, American Society of Anesthesiologists physical status; CCI, Charlson comorbidity index.

## Data Availability

The data are contained within the article.
